# The virtual hand illusion is moderated by context-induced spatial reference frames

**DOI:** 10.3389/fpsyg.2015.01659

**Published:** 2015-10-28

**Authors:** Jing Zhang, Ke Ma, Bernhard Hommel

**Affiliations:** ^1^Cognitive Psychology Unit, Institute for Psychological Research and Leiden Institute for Brain and Cognition, Leiden UniversityLeiden, Netherlands; ^2^Center for the Study of Language and Cognition, Zhejiang UniversityHangzhou, China

**Keywords:** body image, self-recognition, sense of ownership, virtual hand illusion, spatial reference frame

## Abstract

The tendency to perceive an artificial effector as part of one’s own body is known to depend on temporal criteria, like the synchrony between stimulus events informing about the effector. The role of spatial factors is less well understood. Rather than physical distance, which has been manipulated in previous studies, we investigated the role of relative, context-induced distance between the participant’s real hand and an artificial hand stimulated synchronously or asynchronously with the real hand. We replicated previously reported distance effects in a virtual reality setup: the perception of ownership increased with decreased distance, and the impact of synchrony was stronger for short distances. More importantly, we found that ownership perception and impact of synchrony were affected by previous distance: the same, medium distance between real and artificial hand induced more pronounced ownership after having experienced a far-distance condition than after a near-distance condition. This suggests that subjective, context-induced spatial reference frames contribute to ownership perception, which does not seem to fit with the idea of fixed spatial criteria and/or permanent body representations as the sole determinants of perceived body ownership.

## Introduction

How do we perceive ourselves and what are the mechanisms underlying our ability to perceive our body as constituting our bodily self? A recent technique to investigate this issue is the rubber hand illusion (RHI) and its virtual-reality version, the virtual hand illusion (VHI). In the RHI/VHI, participants perceive an artificial physical or virtual hand as a part of their own body (Botvinick and Cohen, 1998; Ehrsson et al., 2004; Tsakiris and Haggard, 2005; Slater et al., 2008; Shimada et al., 2009). This illusion can be induced by synchronously stroking a rubber/virtual hand placed in front of a participant in such a way that it seems extend from the participant’s body, while the corresponding real hand is hidden from view. After a short while of synchronous stroking or, as in the virtual case, of perceived synchrony between own and artificial hand, the participant starts to get the perceptual impression that the rubber/virtual hand becomes his or her own hand.

Temporal synchrony between multimodal input coming from the real and artificial hand is crucial for the illusion, as asynchronous conditions (in which one stimulus stream is delayed with respect to the other by several 100s of ms) commonly produce significantly lower ownership ratings. Interestingly, however, there is also evidence for spatial criteria for perceived ownership. While the illusion is most pronounced with minimal gaps between real and artificial hand (e.g., Costantini and Haggard, 2007; Lloyd, 2007; Gentile et al., 2013), the illusion does survive some discrepancies. For example, Lloyd (2007) showed that the strength of the illusion declined significantly if the rubber hand is placed horizontally more than 27.5 cm away from the participant’s real corresponding hand. However, Zopf et al. (2010) did not find a reduction in RHI strength with distances up to 45 cm between the real and fake hands, which might suggest that the illusion relies on reaching distance. Preston (2013) considered the possibility that it may not be the absolute distance between real and artificial hand that matters but, rather, the distance between real hand and trunk. She manipulated both the absolute distance between real and artificial hand and their relative distance from body midline. The finding is that the strength of the illusion is reduced only if the artificial hand is far from both the real hand and the trunk. Kalckert and Ehrsson (2014) varied the vertical instead of the horizontal distance. The illusion became weaker with increasing distance.

These and related findings were taken to suggest a role of spatial reference frames when considering whether an artificial hand is or is not part of one’s body. Maravita et al. (2003) proposed that although visuotactile interactions are usually most pronounced for stimuli near the real body part, the space to be considered can be plastically modified with active tool-use. If so, the ownership-related spatial reference frame could be flexible. FMRI studies already showed some evidence for this possibility. Brozzoli et al. (2012) found that the hand-centered encoding of space was remapped when a rubber hand was perceived as one’s own. In the present study, we were interested to see whether the situational context might also affect the spatial reference frame used to determine body ownership. The reasons for considering this possibility were some informal observations in other studies from our lab, where the order or presence/absence of conditions seemed to play a role (e.g., see Zhang and Hommel, 2015). Consider, for instance, a condition in which real hand (and body) and artificial hand are separated by a noticeable spatial gap. After just having experienced a condition with a closer connection between real and artificial hand, the artificial hand may now be perceived as rather distant, and the perception of ownership may be reduced. In contrast, after just having experienced a condition with an even greater gap between real and artificial hand, the artificial hand may now be perceived as rather closely connected to one’s real hand or body and, thus, motivate rather high ownership ratings.

We tested this possibility by presenting participants with a condition with a noticeable but not extreme gap between real and artificial hand after having them presented with an even larger or with a smaller gap. That is, we used far-distance and near-distance conditions as *priming* conditions and a medium-distance condition as *test* condition. We used a VHI setup, in which participants wore a data glove and were presented with a 3D virtual hand. Tactile stimulation was applied through vibrators attached to the data glove, which avoids the rather artificial stroking procedure required for the traditional RHI setup. Given previous reports about divergent findings for different kinds of ownership-perception indicators (Rohde et al., 2011), we used the standard ownership questionnaire (adapted for the virtual setup), in addition to proprioceptive drift and skin conductance response (SCR), two commonly used “objective” measures to assess the ownership illusion. Our prediction was that the same medium-distance test condition should produce lower ownership ratings after a near-distance priming condition than after a far-distance priming condition.

## Materials and Methods

### Participants

There were 34 participants (three more were tested but did not complete the experiment), all of them were student volunteers (eight males; mean age = 23 years, *SD* = 2.38, range 18–28) from Leiden University, unfamiliar with the rubber/VHI, who participated in exchange for course credit or pay. Ethical approval for this study was obtained from the local Psychology Research Ethics Committee, and written informed consent was obtained from all participants.

### Design

We used a 2-factorial within-participants design. The two factors were synchrony (synchronous vs. asynchronous) and distance-condition sequence (near-medium vs. far-medium). To avoid the influence of fatigue and response strategies, we divided the experiment into two sessions performed on different days (with 1.32 days on average in between). In the near-medium session, participants were exposed to a condition with a medium-sized gap between their real hand and a virtual hand on the screen in front of them after having been exposed to a condition with a small-sized gap. In the far-medium session, participants were exposed to the same medium-sized gap condition after having been exposed to a condition with a large-sized gap. All participants served in both sessions. Half of the participants participated in the near-medium session before the far-medium session while the other half participated in reversed order. In each of the two distance conditions per session, the participant would be exposed to a synchronous condition and an asynchronous condition. The order of these two synchrony conditions was the same for the two distance conditions for a given participant, but the order of synchronous and asynchronous conditions was balanced across participants.

### Experimental Setup

The study was performed in a virtual reality environment. The setup consisted of a data glove (Cyberglove, measurement frequency = 100 Hz, latency = 10 ms), virtual reality software (Vizard), and a large projection screen of 212 cm × 133 cm, which was around 50 cm away from the participants. The Cyberglove had a vibrator on the palm, through which we were able to apply the tactile stimulation (vibration frequency = 0–125 Hz). Participants wore the glove on their right hand, which during the experiment was placed in a fixed position inside a black box (50 cm × 24 cm × 38 cm) with the palm facing up. A Biopac MP100 acquisition unit and AcqKnowledge software were used for the SCR data recording.

**FIGURE 1:**
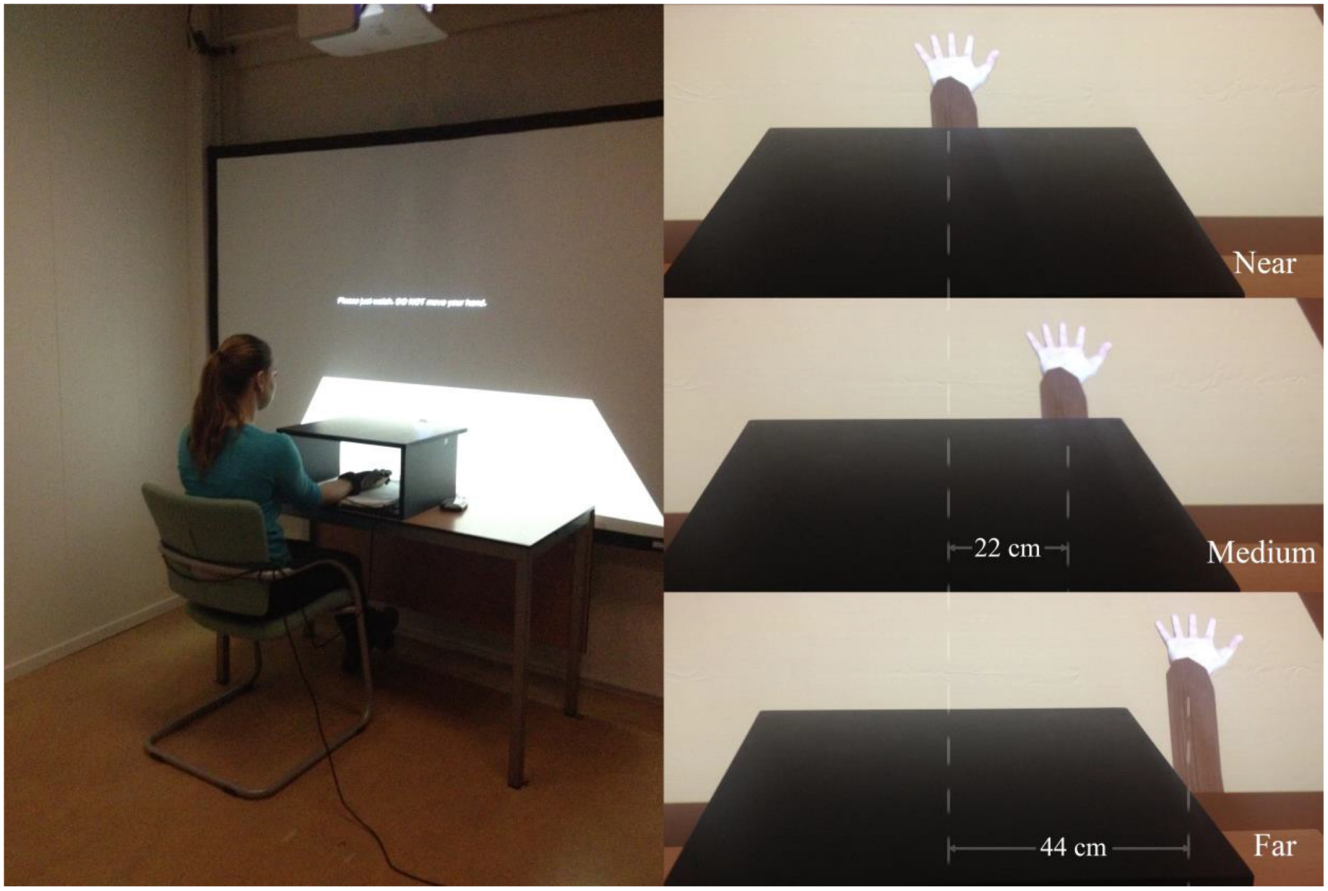
**The experimental setup **(left)** and the three different positions at which the virtual hand was shown on the screen (right)**.

We used a virtual hand from Vizard character set and imported the tracker and data glove module into Vizard. The virtual hand was projected on the large screen in three different positions (always aligned with the participant’s real hand): near (seemingly extending from the real hand), medium (22 cm horizontally away from the near position), and far (44 cm horizontally away from the near position), as shown in **Figure 1**. In the near conditions, the virtual hand was projected in alignment with the participant’s real hand, which looked as if the virtual hand extended from the real hand; and in the far conditions, the virtual hand was 44 cm horizontally away from the near position.

### Measurements

Subjective ownership perception was assessed by means of the standard ownership questionnaire developed by Botvinick and Cohen (1998), which we only adjusted to the virtual setup. Corresponding versions of this questionnaire have been used in various kinds of rubber/VHI experiments (Botvinick and Cohen, 1998; Makin et al., 2008; Zhang and Hommel, 2015). We also considered more objective measures for explorative purposes, namely, proprioceptive drift (Longo et al., 2008; Kammers et al., 2009; Riemer et al., 2013; Ma and Hommel, 2015b), and SCR (Armel and Ramachandran, 2003; Yuan and Steed, 2010; Ma and Hommel, 2013, 2015a,b). Subjective and objective measures have shown different outcomes in various cases (e.g., Ma and Hommel, 2013, 2015a), suggesting that they do not reflect the exact same mechanisms, and objective measures such as proprioceptive drift have been criticized for several reasons (Rohde et al., 2011; Folegatti et al., 2012). This makes it difficult to make predictions for the more objective measures, but we nevertheless analyzed and report effects for all three measures.

#### Questionnaire

We used an adapted version (Slater et al., 2008; Padilla et al., 2010; Ma and Hommel, 2013) of the standard nine-item questionnaire (Botvinick and Cohen, 1998) to assess the strength of ownership illusion in our design. Q1–Q5 are related to the experience of perceiving the hand as one’s own (Kalckert and Ehrsson, 2014; Ma and Hommel, 2015a,b), and Q6–Q9 assess possible side effects of the illusion. Each statement was scored on a 7-point Likert scale, ranging from 1 for “strongly disagree” to 7 for “strongly agree”, and 4 for ‘uncertain.’ The questionnaire items are shown below:

Q1: I felt as if I was looking at my own hand.
Q2: I felt as if the virtual hand were my hand.
Q3: It seemed as if I were feeling the touch of the ball in the location where I saw the virtual hand touched.
Q4: It seemed as though the touch I felt was caused by the ball touching the virtual hand.
Q5: I felt as if the virtual were part of my body.
Q6: It felt as if my (real) hand were drifting toward the virtual hand on the screen.
Q7: It seemed as if I might have more than one right hand or arm.
Q8: I felt as if my real hand were turning virtual.
Q9: I felt as if my right hand had disappeared.

So far, no psychometrically analyzed version of the questionnaire has been developed and no absolute criteria for determining the absence or presence of an illusion have been suggested. We therefore used the comparison between synchronous and asynchronous conditions as a proxy. A significantly stronger ownership score in synchronous as compared to asynchronous conditions was thus taken to indicate a relative increase in perceived ownership, and the size of the increase was taken to reflect the strength of the impact of the corresponding factor.

#### Proprioceptive Drift

The method we used for the proprioceptive drift measurement was the same as in our earlier study (Ma and Hommel, 2015b). We presented an array of letters on the screen and asked participants to verbally report the felt location of their real right middle finger by choosing the particularly corresponding letter. To work against response strategies, the letters in the strings were presented in random order. The letter size differed depending on their alphabetic shape, with the biggest letter measuring approximately 2 cm. We recorded the corresponding letter before and after the illusion induction process (Botvinick and Cohen, 1998; Tsakiris and Haggard, 2005; Kalckert and Ehrsson, 2014). We calculated the distance between the letters and the screen side, and calculated the proprioceptive drift by subtracting the distance in the post-measure from the distance in pre-measure, so that positive values imply a drift toward the virtual hand.

#### SCR

The method we used for the SCR measurement was also the same as our earlier study (Ma and Hommel, 2015b). We measured SCR during a threat phase, in which a virtual knife appeared above the virtual hand on the screen and moved down to cut the virtual hand. It took 4 s to cut the virtual hand and another 4 s to move back to the original position. The cutting procedure was repeated five times. We defined a latency onset window between 1 and 6 s after stimulus/event onset, namely, when the virtual knife cut the virtual hand, with the skin conductivity level before event onset serving as baseline (see Boucsein, 1992; Figner and Murphy, 2010; Ma and Hommel, 2013, 2015a,b). We then calculated the magnitude of the event-induced SCR by subtracting baseline skin conductivity from the peak amplitude of the SCR during the analyzed time window.

### Procedure

When participants arrived in the lab, they were asked to put the glove on their right hand and a SCR remote transmitter on their left wrist with a strap. Then they were seated in front of a desk and a projection screen (see **Figure 1**). They were instructed to put their right hand with palm upward into a box in between the participant and the screen, so they could not see their own right hand. Participants’ right hands were placed at the middle position of the box, and they were asked not to move their right hand during the experiment.

As mentioned already, each session consisted of four blocks (e.g., far/synchronous, far/asynchronous, medium/synchronous, medium/asynchronous). The sequence of events was the same for each block. First, participants judged the location of the right middle finger of their real hand, as described above. Second, the illusion was induced by means of visuotactile stimulation. The virtual hand was shown on the screen, seen as extending from the participant’s right hand, and a small virtual ball appeared above the virtual hand. The ball took 4 s to move down to contact the virtual hand’s palm, and then took another 4 s to return to its original position; this illusion induction procedure was repeated for 90 s. In the synchronous conditions, the contact between the virtual ball and hand was associated with the onset of the palm vibration stimulator of the glove, so as to apply synchronous visuotactile stimulation. In the asynchronous conditions, the vibration was delayed by 4 s, so that visual and tactile stimulation did not match. The vibration lasted for 1 s for every ball movement procedure in all conditions. Third, participants would again judge the location of their real right middle finger, and then fill in the ownership questionnaire on paper with his/her their unstimulated hand. Fourth, the same illusion induction procedure in the second step was implemented again, and then the virtual ball was replaced by a virtual knife, the threat phase started, SCR was measured while the virtual hand was threatened by the virtual knife, as described above. Finally, participants were asked to take a short break before they experienced the next block.

## Results

### Priming Conditions (Near and Far)

All questionnaire items scores for priming conditions were submitted to 2 × 2 ANOVA with the factors synchrony (synchronous vs. asynchronous) and distance (near vs. far). Means and standard errors for each question item in each condition, F, P and effect size values for each question item, are shown in **Table 1**. The synchrony pattern of results is similar to previous studies (Botvinick and Cohen, 1998; Slater et al., 2008), ownership questions (Q1–Q5) showed significant synchrony effects, while control questions (Q6–Q9) did not (except for Q8).

**Table 1:** Priming conditions (near and far): means (M) and standard errors (SE); F, P, and effect size values for all the questionnaire items scores, and also for the aggregate scores of Q1–Q5, with *df* = 33.

M/SE	Q1	Q2	Q3	Q4	Q5	Q6	Q7	Q8	Q9	Q1–Q5
Near-synchronous	4.35/0.27	4.38/0.26	5.32/0.19	4.88/0.24	4.00/0.23	3.32/0.26	2.23/0.19	3.00/0.26	1.19/0.15	4.59/0.19
Near-asynchronous	3.09/0.25	2.59/0.19	2.53/0.27	1.94/0.21	2.62/0.21	2.88/0.23	2.15/0.19	2.47/0.22	1.85/0.18	2.55/0.16
Far-synchronous	3.18/0.27	3.35/0.29	4.79/0.27	4.09/0.29	2.91/0.26	2.94/0.25	2.15/0.22	2.44/0.25	1.97/0.17	3.66/0.23
Far-asynchronous	2.73/0.28	2.79/0.24	2.59/0.28	2.06/0.22	2.41/0.23	2.71/0.26	1.82/0.17	2.29/0.21	1.88/0.21	2.52/0.19
*F/p/*$\eta_{p}^{2}$ (Distance)	11.10/0.002/0.25	3.35/0.076/0.09	1.30/0.262/0.03	2.52/0.122/0.07	11.67/0.002/0.26	1.75/0.195/0.05	1.64/0.210/0.05	4.51/0.041/0.12	0.17/0.681/0.01	9.84/0.004/0.23
*F/p/*$\eta_{p}^{2}$ (Synchrony)	16.24/<0.001/0.33	30.79/<0.001/0.48	76.22/<0.001/0.70	93.98/<0.001/0.74	23.45/<0.001/0.41	2.80/0.104/0.08	2.16/0.151/0.06	5.74/0.022/0.15	0.48/0.492/0.01	71.47/<0.0010.68
*F/p/*$\eta_{p}^{2}$ (Interaction)	6.29/0.017/0.16	11.720.002/0.26	2.45/0.127/0.07	5.59/0.024/0.14	5.34/0.03/0.14	0.53/0.471/0.02	0.55/0.462/0.02	1.05/0.313/0.03	0.01/0.905/0.01	18.81/<0.0010.36

**FIGURE 2:**
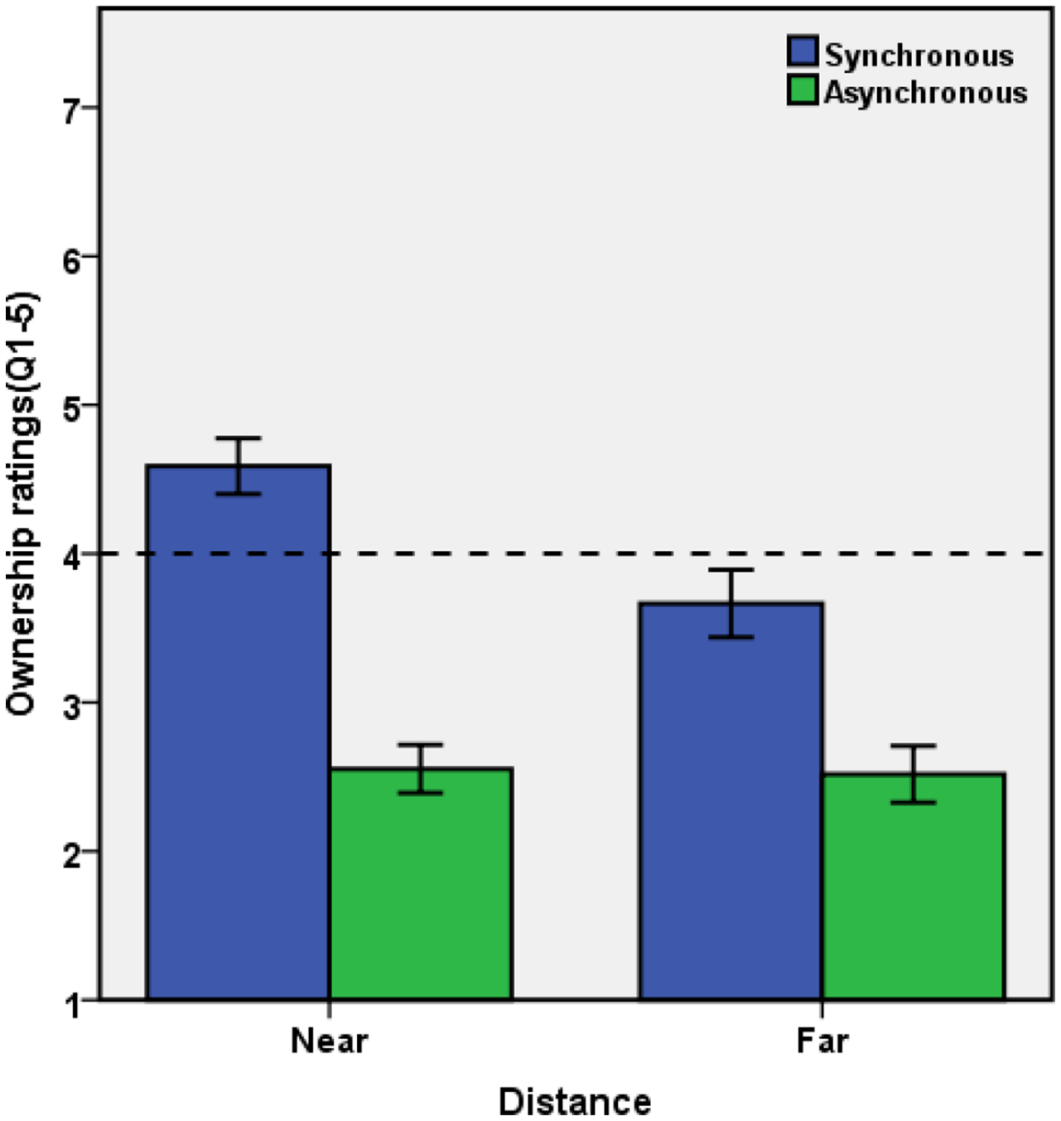
**Mean Score of Ownership Questions (Q1–Q5) for priming condition.** Error bars represent ±1 SE.

Following Kalckert and Ehrsson (2014) and Ma and Hommel, 2015a,b), we aggregated the ownership questions (Q1–Q5) and computed their mean to represent sense of ownership. This score was analyzed by means of a 2 × 2 ANOVA with the factors synchrony (synchronous vs. asynchronous) and distance (near vs. far). There were significant main effects of synchrony, *F*(1,33) = 71.470, *p* < 0.001, $\eta_{p}^{2}$ = 0.684, indicating a stronger sense of body ownership for synchronous visuotactile stimulation (*M* = 4.126, *SE* = 0.180) than for asynchronous stimulation (*M* = 2.535, *SE* = 0.159); and of distance, *F*(1,33) = 9.837, *p* = 0.004, $\eta_{p}^{2}$ = 0.230, showing a stronger sense of body ownership for near (*M* = 3.571, *SE* = 0.134) than for far (*M* = 3.091, *SE* = 0.184) placement of the virtual hand. Importantly, the interaction between the two factors was also significant, *F*(1,33) = 18.812, *p* < 0.001, $\eta_{p}^{2}$ = 0.363, indicating that the synchrony effect was more pronounced for the near than the far condition, see **Figure 2**. Two tailed paired *t*-tests revealed that the synchrony effect was significant in both near and far positions, *t*(33) = 8.980, *p* < 0.001, *d* = 1.995, and *t*(33) = 5.703, *p* < 0.001, *d* = 0.943, respectively; and the distance effect was significant in synchronous conditions, *t*(33) = 4.425, *p* < 0.001, *d* = 0.764, but not in asynchronous conditions, *t*(33) = 0.227, *p* = 0.822, *d* = 0.034.

### Test Condition (Medium)

#### Questionnaire

All questionnaire items scores for the test condition were submitted to 2 × 2 ANOVA with the factors synchrony (synchronous vs. asynchronous) and context (near-medium vs. far-medium). Means and standard errors for each question item in each condition, F, P, and effect size values for each question item, are shown in **Table 2**.

**Table 2:** Test conditions (near-medium and far-medium): means (M) and standard errors (SE); F, P, and effect size values for all the questionnaire items scores, and also for the aggregate scores of Q1–Q5, with *df* = 33.

M/SE	Q1	Q2	Q3	Q4	Q5	Q6	Q7	Q8	Q9	Q1–Q5
Near-medium-synchronous	3.00/0.25	3.56/0.28	4.03/0.29	4.21/0.28	3.32/0.26	3.47/0.27	2.06/0.18	2.73/0.24	2.21/0.19	3.62/0.22
near-medium-asynchronous	2.71/0.25	2.73/0.23	2.35/0.24	2.09/0.19	2.56/0.20	2.88/0.26	2.23/0.21	2.53/0.21	1.97/0.19	2.49/0.18
far-medium-synchronous	4.41/0.23	4.47/0.22	5.23/0.17	4.94/0.21	4.12/0.23	3.38/0.28	2.29/0.20	3.09/0.25	2.21/0.24	4.63/0.17
far-medium-asynchronous	3.15/0.26	3.15/0.29	2.94/0.28	2.44/0.25	2.82/0.22	2.88/0.24	2.12/0.22	2.41/0.25	2.00/0.12	2.90/0.21
*F/p/*$\eta_{p}^{2}$ (Context)	23.45/<0.001/0.41	9.29/0.005/0.22	23.22/<0.001/0.41	12.45/0.001/0.27	9.84/0.004/0.23	0.05/0.817/0.01	0.11/0.743/0.01	0.33/0.571/0.01	0.01/0.929/<0.01	39.82/<0.0010.55
*F/p/*$\eta_{p}^{2}$ (Synchrony)	10.23/0.003/0.24	21.71/<0.001/0.40	69.92/<0.001/0.68	105.84/<0.001/0.76	27.35/<0.001/0.45	8.07/0.008/0.20	01.000/0	8.96/0.005/0.21	2.36/0.134/0.07	67.00/<0.0010.67
*F/p/*$\eta_{p}^{2}$ (Interaction)	7.29/0.011/0.18	1.77/0.193/0.05	2.67/0.111/0.07	1.30/0.262/0.04	3.13/0.086/0.09	0.08/0.779/0.01	2.42/0.129/0.07	2.81/0.103/0.08	0.01/0.908/< 0.01	7.19/0.011/0.18

**FIGURE 3:**
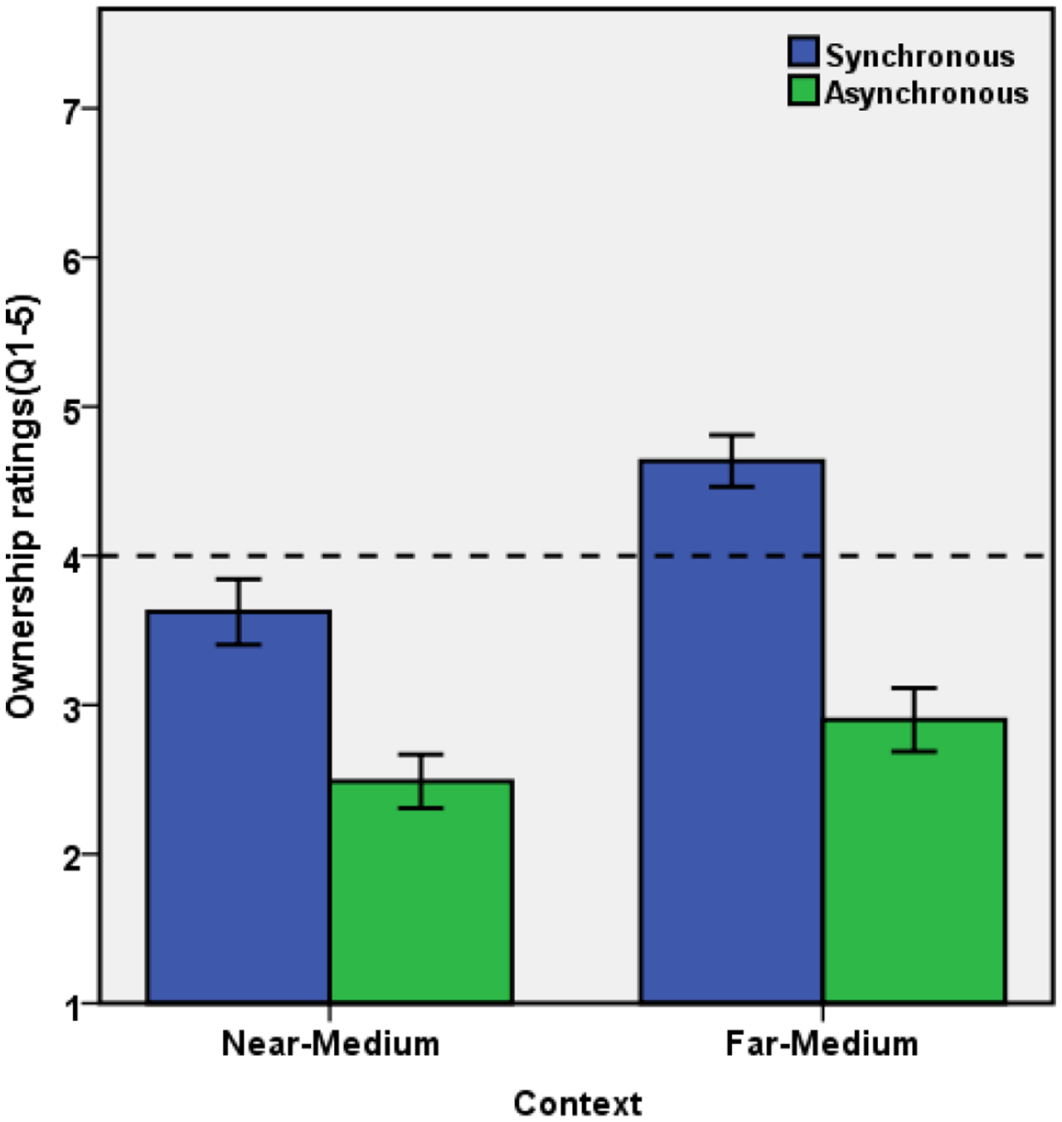
**Mean Score of Ownership Questions (Q1–Q5) for test condition.** Error bars represent ±1 SE.

The mean score for ownership (Q1–Q5) was analyzed by means of a 2 × 2 ANOVA with the factors synchrony (synchronous vs. asynchronous) and context (near-medium vs. far-medium). There were significant main effects of synchrony, *F*(1,33) = 67.002, *p* < 0.001, $\eta_{p}^{2}$ = 0.670, showing a stronger sense of ownership for synchronous (*M* = 4.129, *SE* = 0.175) than for asynchronous conditions (*M* = 2.694, *SE* = 0.187); and of context, *F*(1,33) = 39.818, *p* < 0.001, $\eta_{p}^{2}$ = 0.547, showing stronger ownership for the far-medium (*M* = 3.768, *SE* = 0.156) than the near-medium condition (*M* = 3.056, *SE* = 0.179). The interaction between the two factors was also significant, *F*(1,33) = 7.192, *p* = 0.011, $\eta_{p}^{2}$ = 0.179, suggesting that the synchrony effect was more pronounced in the far-medium than the near-medium condition, see **Figure 3**. Two tailed paired *t*-tests showed that the synchrony effect was significant in the near-medium [*t*(33) = 6.271, *p* < 0.001, *d* = 0.974] and the far-medium condition [*t*(33) = 7.485, *p* < 0.001, *d* = 1.538]; and the context effect was significant in both synchronous conditions, *t*(33) = 5.458, *p* < 0.001, *d* = 0.882, and asynchronous conditions, *t*(33) = 3.244, *p* = 0.003, *d* = 0.359.

#### Proprioceptive Drift

The proprioceptive drift results were log transformed and the normality of distribution was determined using the Shapiro–Wilk test, *p* > 0.8.

**FIGURE 4:**
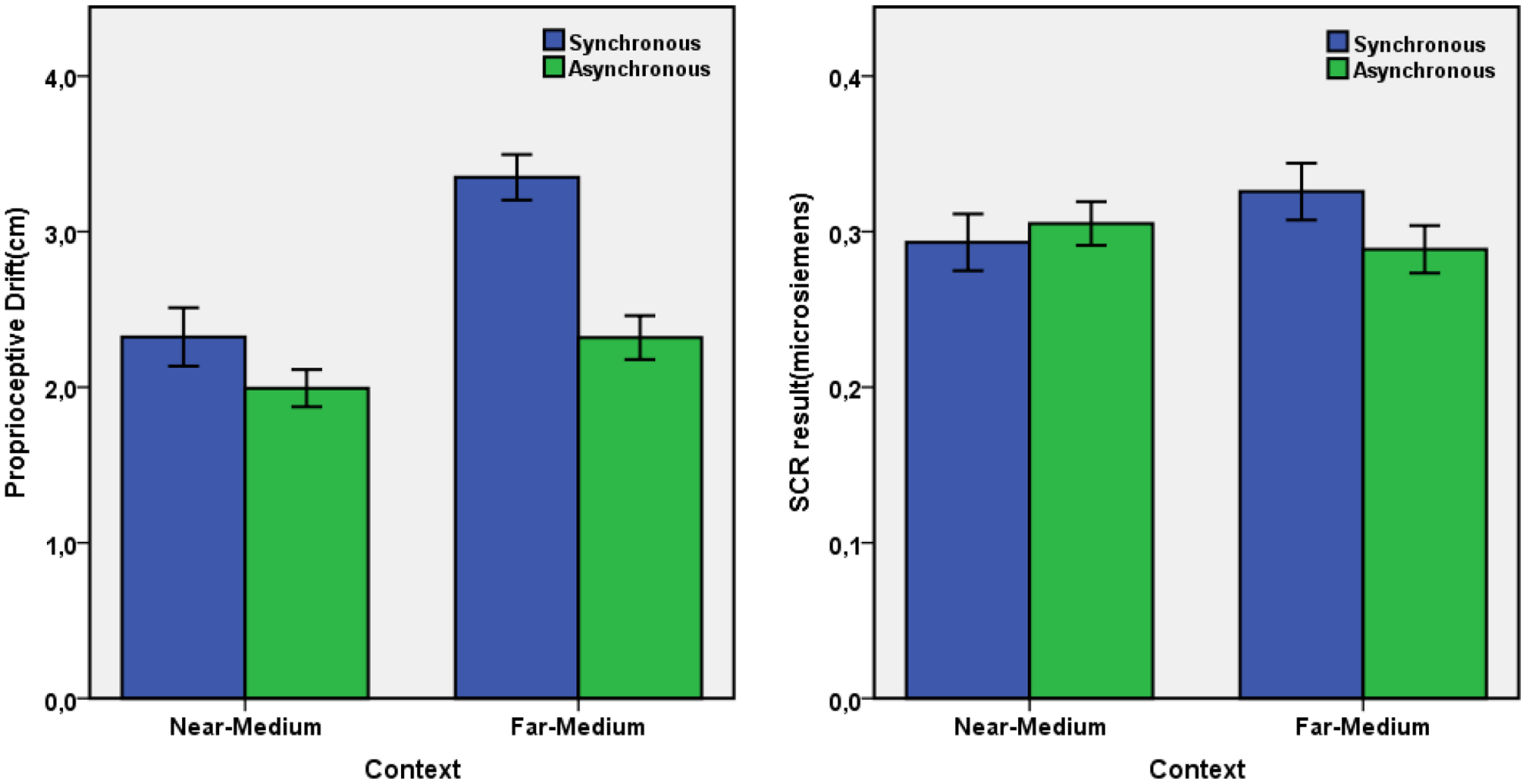
**Proprioceptive Drift **(left)** and SCR **(right)** results, the error bars represent ±1 SE**.

The transformed scores of proprioceptive drift for each condition were submitted to a 2 × 2 ANOVA with the factors synchrony (synchronous vs. asynchronous) and context frame (near-medium vs. far-medium). There were significant main effects of synchrony, *F*(1,33) = 26.035, *p* < 0.001, $\eta_{p}^{2}$ = 0.441, showing a stronger proprioceptive drift with synchronous (*M* = 2.836 cm, *SE* = 0.107) than asynchronous stimulation (*M* = 2.156 cm, *SE* = 0.100); and of context, *F*(1,33) = 24.804, *p* < 0.001, $\eta_{p}^{2}$ = 0.429, showing a stronger proprioceptive drift in the far-medium (*M* = 2.834 cm, *SE* = 0.104) than the near-medium condition (*M* = 2.158 cm, *SE* = 0.105). The interaction also reached significance, *F*(1,33) = 4.170, *p* = 0.049, $\eta_{p}^{2}$ = 0.112, indicating that the synchrony effect was more pronounced in the far-medium than the near-medium condition. As shown in **Figure 4**, the outcome pattern was comparable to that for the ownership questionnaire items. Two tailed paired *t*-tests showed that the synchrony effect was significant in the far-medium [*t*(33) = 5.180, *p* < 0.001, *d* = 1.229], but not the near-medium condition [*t*(33) = 1.412, *p* = 0.167, *d* = 0.368]; and the context effect was significant in synchronous conditions, *t*(33) = 3.954, *p* < 0.001, *d* = 1.054; but not in asynchronous conditions, *t*(33) = 1.941, *p* = 0.061, *d* = 0.429.

#### SCR

The SCR results were log transformed and the normality of distribution was determined using the Shapiro–Wilk test, *p* > 0.6.

The transformed scores of SCR for each conditions were submitted to a 2 × 2 ANOVA with the factors synchrony (synchronous vs. asynchronous) and context (near-medium vs. far-medium). There was no main effect but the interaction was significant, *F*(1,33) = 5.667, *p* = 0.023, $\eta_{p}^{2}$ = 0.147, indicating that the synchrony effect was more pronounced in the far-medium than the near-medium conditions (see **Figure 4**). Two-tailed paired *t*-tests revealed that the synchrony effect was significant for the far-medium condition, *t*(33) = 2.587, *p* = 0.014, *d* = 0.379, but not for the near-medium condition, *t*(33) = 0.723, *p* = 0.475, *d* = 0.128; while the context effect was not significant in synchronous conditions, *t*(33) = 1.821, *p* = 0.078, *d* = 0.306, or asynchronous conditions, *t*(33) = 1.135, *p* = 0.265, *d* = 0.194.

## Discussion

Temporal relationships between different sources of intermodal stimulation are known to affect the degree of perceived body ownership. Spatial factors also play a role, but they are less well understood. In contrast to previous studies, which looked into the distance between real and artificial hands, we tested the possibility that the situational context has an impact on whether a given distance is perceived as short or long. We thus tested the same medium-distance condition after a near-distance and after a far-distance condition, to see whether ownership ratings are more pronounced in the latter than in the former condition. Our findings provide clear evidence that the situational context affects perceived ownership. In particular, our findings have three implications.

Firstly, the questionnaire results for the two priming conditions showed that we were able to replicate the distance effect reported by Lloyd (2007) in a virtual setup. When the virtual hand was placed in a near position, questionnaire scores were significantly higher than those in the far position. It is interesting to see that the absolute ownership scores were not very high in the present study, probably because our setup made the virtual hand look a little bit far away from the participants even in the near condition. This is also consistent with previous studies (Lloyd, 2007; Preston, 2013), which suggested important roles of both distance and reaching space. Hence, our findings can be taken to confirm that distance effects are rather robust.

Second, our results showed that the synchrony-induced increase in ownership perception was significantly stronger for a near than far placed virtual hand. This provides even more direct evidence for the idea that ownership perception takes the distance between real and artificial hand (and/or between real body and artificial hand) into account. This is consistent with previous observations and corresponding theoretical claims (Lloyd, 2007; Tsakiris, 2010; Preston, 2013; Kalckert and Ehrsson, 2014). As Tsakiris (2010) suggested, one criterion for the ownership perception may occur as a result of the comparison between current sensory input and body-related reference frames. Alternatively, a distance rule may apply. Such a rule may operate continuously, with the probability of ownership perception increasing with decreasing distance, discontinuously, with ownership perception being restricted to candidate effector is within reaching space, or reflect some interaction of both. Given that we observed interactions between distance and synchrony for both (near and far) priming conditions and (medium) test conditions, a merely discontinuous rule does not seem to be sufficient: given that all our conditions fell into reaching space, such a simple rule could not account for such interactions. This leaves a simple distance rule and an interaction between a distance rule and a discontinuous criterion as possibilities.

Third, in the test condition, perceived body ownership was affected by the perceptual context: While absolute distance was kept constant, the relative size of the ownership illusion varied as a function of the context-induced relative distance between real hand (or body) and artificial hand. Given the impact of actual distance observed in the priming conditions, this should not be taken to rule out contributions from physical distance. However, relative distance that relates previous experiences to the current distance between real and artificial hand seems to contribute as well. This observation is not consistent with the assumption that ownership perception relies on objective situational variables and internal representations thereof alone. It also does not fit with assumptions that only objective spatial parameters, like reaching space, and/or stable pre-existing body models play a role. Rather, ownership perception seems to rely on various informational sources that include subjective impressions informed by previous experiences in the same situation (Ma and Hommel, 2015b).

One thing to note is that, in our experiment setup, the virtual hand seemed to extend from the participant’s real hand into the screen, so that the virtual hand always looked longer than the real hand. Could that have affected our results? Even though we are unable to exclude main effects, there are two reasons why we do not consider it plausible that this aspect can account for our main observations. For one, the “virtual extension” was the same in all conditions, as we only manipulated the horizontal distance between the real and the artificial hand. This suggests that, even if there was some effect, it should have impacted all conditions equally. For another, previous RHI studies suggest that such kinds of “virtual extensions” do not seem to influence the synchrony effect significantly. For example, in Preston and Newport (2012), the experimenter pulled the participant’s arm while participants viewed the pull in a real-time video of themselves. In the video, the arm looked like being stretched to twice of its normal length. Participants did have the impression of their arm being stretched and they overestimated reaching distance, but the actual reaches were unaffected. In one of Armel and Ramachandran’s (2003) experiments, the arm looked like being stretched to 0.91 m, but the basic illusion was still obtained. Finally, Kilteni et al. (2012) found that participants experienced ownership illusions even for a virtual arm that was about three times as long as a real arm. As we mentioned before, the ownership scores in the present study are relatively low, an observation that we attribute to the arm extension design we used in our study. Similar observations have been made in previous studies (Armel and Ramachandran, 2003; Kilteni et al., 2012), where ownership ratings were relatively low when the rubber/virtual hand seem to be much longer than the real arm.

In addition to the more theoretical implications, our observations are also of relevance methodologically. For one, they strongly suggest that sequence effect can play an important role in moderating the size of the ownership illusion. Our findings also provide convergent evidence for the conclusion that ownership questionnaires, proprioceptive drift, and SCR are not fully equivalent methods to assess perceive body ownership. In the present study, the questionnaire turned out to be much more sensitive to the impact of our manipulations on self-perception than the other two measures, which fits with previous observations (Rohde et al., 2011; Folegatti et al., 2012; Ma and Hommel, 2013, 2015a).

## Conclusion

The present study extends our knowledge about the cognitive process underlying RHI/VHI by demonstrating the flexibility of spatial criteria for moderating perceived body ownership. This adds to previous evidence that ownership perception may not be a simple function of continuous or discontinuous distance rules or a cross-situationally stable body image. Rather, there is increasing evidence that multiple sources of information contribute to the illusion, so that the relative importance of a given source may very well depend on the situation and the existence of other informational sources. This again is consistent with previous claims that body representations are dynamic and continuously updated to reflect the present situation (e.g., Graziano and Botvinick, 2002; Ehrsson, 2012).

## Conflict of Interest Statement

The authors declare that the research was conducted in the absence of any commercial or financial relationships that could be construed as a potential conflict of interest.
